# Physical Activity, Lifestyle Factors and Oxidative Stress in Middle Age Healthy Subjects

**DOI:** 10.3390/ijerph15061152

**Published:** 2018-06-01

**Authors:** Elisabetta Carraro, Tiziana Schilirò, Felicina Biorci, Valeria Romanazzi, Raffaella Degan, Daniela Buonocore, Manuela Verri, Maurizia Dossena, Sara Bonetta, Giorgio Gilli

**Affiliations:** 1 Department of Public Health and Pediatrics, University of Torino, Piazza Polonia 94, 10126 Torino, Italy; elisabetta.carraro@unito.it (E.C.); valeria.romanazzi@gmail.com (V.R.); raffaella.degan@unito.it (R.D.); sara.bonetta@unito.it (S.B.); giorgio.gilli@unito.it (G.G.); 2 Center of Sport and Preventive Medicine, University of Torino, Piazza Bernini 12, 10143 Torino, Italy; felicina.biorci@unito.it; 3 Department of Biology and Biotechnology “L. Spallanzani”, University of Pavia, Via Ferrata 9, 27100 Pavia, Italy; daniela.buonocore@unipv.it (D.B.); manuela.verri@unipv.it (M.V.); maurizia.dossena@unipv.it (M.D.)

**Keywords:** physical activity, exercise training, lifestyle, dietary habits, oxidative stress, public health

## Abstract

Oxidative stress (OS) has been recognized to play a primary role in many acute and chronic diseases. Environmental and lifestyle factors, such as physical activity and dietary intake are involved in the oxidative balance, but their specific influence remains unclear. In order to contribute to a greater characterization of the oxidative status in relation to exercise training and to environmental and lifestyle factors, different biomarkers—pro-oxidant capacity (d-ROMs), anti-oxidant capacity (BAP), radical scavenging activity (DPPH) and DNA damage (8-OHdGuo)—were measured in biological samples of a group of healthy middle aged subjects. The evaluation of the investigated biomarkers highlighted a significant effect of exercise training on OS, measured as d-ROMs and 8OhdGuo, in subjects playing regular physical activity. An association of the OS status measured by DPPH and 8-OhdGuo with the condition of living in urban high traffic areas was also found. Otherwise dietary habits did not reveal any significant effect on OS levels by the investigated biomarkers. As a whole the results obtained in this investigation suggested that a correct lifestyle, with regular physical activity practice, contributes to control the OS status in middle age subjects.

## 1. Introduction

Oxidative stress (OS) is defined as a balance disorder between Reactive Oxygen Species (ROS) and antioxidant defenses. The alterations of the normal redox state of cells can cause toxic effects through the production of free radicals and peroxides that can potentially damage proteins, lipids, and DNA of the cell [1]. It is known that OS plays an important role in the development of most chronic diseases, affecting the health of individuals throughout life [2].

Food habits, environmental pollution and physical activity can play a significant role in the oxidative balance of the organism [3]. Concerning physical activity, the different impacts on OS reported in literature are probably due to the use of different biochemical/cellular markers as well as different physical exercise protocol adoption by the various investigations [4,5]. Variations in type, intensity and duration of the exercise training can activate different patterns of oxidant–antioxidant balance leading to different responses in terms of cellular damage. In fact exercise-induced ROS are transient and have been reported to promote many redox-specific health adaptations [6]. Moreover sedentary behaviour and excess energy intake have been reported to induce chronic levels of ROS resulting in pathological responses such as insulin resistance [7] and chronic lifestyle diseases [2], many of which may be alleviated by regular exercise-induced redox adaptations [6]. High-intensity exercise has been shown to promote similar, and in some cases, greater metabolic health benefits than that of moderate intensity exercise [8,9]. The complexity of the relationship between physical activity and oxidative balance could be explained by the hormesis theory [10,11].

**Figure 1:**
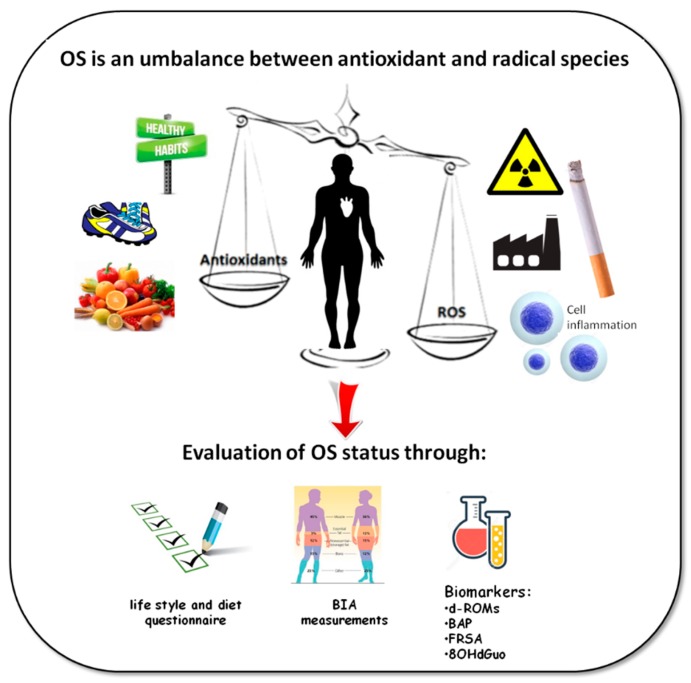
Study design: evaluation of biological markers of OS in a healthy middle age adult group in relation to exercise training, taking into account the influence of environmental and lifestyle factors.

The purpose of the present study was to evaluate the OS status within a healthy middle age adult group in relation to exercise training practice and to environmental and lifestyle factors, considering that between 40–50 years the physiological protection against OS starts to decrease and a not adequate lifestyle could exacerbate and accelerate this process. A series of OS biomarkers was investigated: the total biological antioxidant potential (BAP), the radical oxygen metabolites (d-ROMs), the total free radical scavenging activity (FRAS), the oxidative damage at DNA level in terms of 8-hydroxy-7,8-dihydro-2′-deoxyguanosine (8OHdGuo) [12]. The influence of physical activity, dietary habits and environmental exposures as possible contributors to OS were taken into account (Figure 1).

## 2. Materials and Methods

### 2.1. Subjects Recruiting and Questionnaire

Recruiting operations lasted about 3 months (January–March 2015), and were based on the voluntary participation for subjects who had signed the informed consent. The study was approved by the Ethic Committee of the University of Torino. The exclusion criteria for participants were: (1) being out of the considered age range; (2) having chronic pathologies such as diabetes, cardiovascular diseases, osteoporosis, hypercholesterolemia, hypertension, hyper/hypothyroidism and obesity; (3) using any drugs and/or medications such as anticoagulants, antihypertensive, regulators of glucose metabolism and drugs for chronic pathologies. Subjects ranging from 35 to 55 year-old were recruited and classified as “trained” since they declared to practice regular physical activity at least 3 times/week, or “untrained” since they do not practice physical activities regularly. The training that each subject in the trained group declared playing regularly as sport represented the physical activity considered in the present survey.

To evaluate the contribution of different factors on OS data on frequency of physical activity, smoking habits, occasional drug or food supplement intake, perception of living in an area with traffic-related heavy air pollution, diet habits were collected by a self-administered questionnaire. Specific items based on the Food Frequency Questionnaire by Istituto Superiore di Sanità [13] were dedicated to dietary information on the frequency of food group consumption, including details on oxidant and antioxidant foods, considering standard servings [14] and seasonality. All dietary information was organized on the self-declared weekly frequencies of assumption: never, on average (2–4 times/week) and ever (5–7 times/week). Weight and height were measured and body tissue composition was determined for each participant using a BIA 101 bioelectrical impedance analyzer (Akern, Pontassieve (FI), Italy) with the packaged Bodygram software, to obtain evaluations on body mass index (BMI), body fat mass (FM), water content (TBW) and the phase angle (PA).

### 2.2. Oxidative Status Evaluation

Biological samples were collected from each subject in order to assay the oxidative status in terms of oxidant and antioxidant potential. The study involved two different investigations with OS evaluations: one on the whole group of participants in condition of rest (baseline) (T_rc_) and the second restricted to the trained group taking OS measurements before (T_0_) and at the end (T_1_) of a training session to highlight differences of the OS condition attributable to acute physical activity. Only for trained subjects biological samples were collected at two times.

The global biochemical evaluation of the OS was performed through the Free Radical Analytical System Kit (FRAS-4 Evolvo—Diacron International, Grosseto, Italy), a spectrophotometric technology that allows the evaluation of the pro-oxidant and the antioxidant components using the Radical Oxygen Metabolites Test (d-ROMs Test) and the Biological Antioxidant Potential Test (BAP Test) respectively [15]. As suggested by manufacturer’s instructions, a single whole blood drop obtained with a fingertip puncture ensures the execution of both of the two tests with the provided kits.

In order to estimate the FRAS of human whole saliva, the reduction rate of 1,1-diphenyl-2-picrylhydrazyl (DPPH test) was performed. Subjects were instructed to pump saliva in a collection tube (salivette/sputum, Sarstedt Inc., Nümbrecht, Germany), then saliva samples were processed as previously suggested by Atsumi [16]. Data were presented as micromoles of DPPH radicals scavenged by 1 mL of saliva.

Finally, a urine spot sample was collected in order to quantify the 8-OHdGuo using an immunoassay for the measurement of DNA oxidative damage with a commercial ELISA kit (Cayman Chemical Company, Ann Arbor, MI, USA) [17].

### 2.3. Statistical Methods

A general descriptive analysis of the population was provided, in which impedance measurements were stratified by gender, age, smoking habits and physical activity. The first investigation was focused on the evaluation of the OS in all subjects, comparing biomarker values at rest condition (T_rc_) with impedance data and food frequency intakes. In the second investigation restricted to the participants belonging to the trained group a comparison of the biomarker values before and after the training session (T_0_ vs. T_1_) was performed. Mean concentrations of biological parameters of OS were compared to the variables (i.e., age, physical activity, smoking habits, etc.) by *t*-test and one-way analysis of variance (ANOVA) in order to analyse two or more groups of independent samples respectively; Spearman’s correlations were used to test the associations between the variables. Data on food intake were firstly singularly analysed, then synthetized by a frequency-score in order to better describe the dietary habits of the study population as possible contribution to OS status. Data processing and analysis were performed using the software SPSS V.22 (SPSS Inc., Armonk, NY, USA), considering significant values with *p* < 0.05.

### 2.4. Compliance with Ethical Standards

All procedures involving human participants were in accordance with the ethical standards of the institutional and/or national research committee and with the 1964 Helsinki Declaration and its later amendments or comparable ethical standards.

## 3. Results and Discussion

### 3.1. Oxidative Status at Baseline (T_rc_)

A total of 51 subjects were included in the survey: 63% were males, about the half population declared to live in heavy air polluted traffic zones of Torino and 19.6% were current smokers; the trained group counted 35 subjects and the untrained one 16 subjects.

Impedance measurements (Table 1) resulted for all analyzed parameters (BMI, TBW, PA and FM) within the reference range for all participants [18]. As expected bodily measurements resulted statistically different with ANOVA analysis stratifying the participants by gender, with higher values in males than females, except for FM values that were analogous and, on the whole, low in both sexes. The higher concentration of total body water of males than females could be related to higher values of BMI, as reported also by other authors [19]. The values of PA that is an indicator of the cellular membranes functionality as well as of the nutritional status, ranged from 5° to 7° in both sexes, in line with the reference values for healthy adults [20]. No statistically significant difference was found analysing the body composition measurements of participants by age ranges, smoking habits, and physical activity.

PA was positively related to BMI values (*r* = 0.202 ns) and negatively related to FM (*r* = −0.196 ns); moreover PA value that was lower in women than in men, decreases with age.

Considering the OS status (Table 2), the evaluation of the redox condition at T_rc_ resulted in the normal range: pro-oxidant status measured by d-ROM test was meanly 17.89 ± 4.22 mg H_2_O_2_/dL, when normal range is 20–25 mg H_2_O_2_/dL; antioxidant status measured by BAP test was meanly 2356.92 ± 507.14 µmol/L, when the normal range reflecting antioxidant plasma defence is 2200–2400 µmol/L. Mean values of DPPH in all subjects were 0.07 ± 0.01 mM and 68.93 ± 115.69 ng/mL for 8OHdGuo. Stratifying the values of these biomarkers by gender and age classes, only pro-oxidant status resulted significantly influenced by gender, with a higher pro-oxidant condition in women than in men (ANOVA *p* = 0.006) in agreement with results obtained in previous surveys [21,22].

Comparing untrained and trained subjects at baseline (T_rc_) no statistically significant differences of OS parameters were revealed as measured by the four biomarkers, demonstrating a comparable OS level between the two groups. Differences of oxidative condition measured by DPPH and 8OHdGuo seem to be related to traffic level (Table 2): DPPH and 8OHdGuo mean values were statistically higher in subjects declaring to live in heavy traffic sites than those living in low traffic areas. The increased formation of 8-OHdG with increased levels of traffic airborne pollution exposure found in our study is in accordance with findings of previous similar studies [23,24]. In fact urban air contains pollutants such as metals and polycyclic aromatic hydrocarbons (PAH) able to induce oxidative DNA damage [25]. Moreover it’s well known that urban traffic and particulate matter can cause adverse health effects in terms of induction of OS and inflammation [26,27,28,29].

Finally, no significant association between the redox condition and smoking habits of participants was found. The absence of OS significant differences related to physical activity level and smoking habits could also be due to the numeric imbalance between the groups (16 untrained vs. 35 trained; 10 smokers vs. 41 non-smokers).

The relationships between OS status and impedance measurements (Table 3) reveal an interesting significant correlation between pro-oxidant status measured by d-ROMs and the age of subjects. In literature an increased level of radical is already assessed at the age of 40 and furtherly amplified at the age of 50, when the radical production is accompanied by the decrease of antioxidant defences. Even if the fat percentages measured in the study participants were in the normal range, as expected a significant positive correlation between the pro-oxidant status and FM values was found: in consideration that higher metabolic rate of the fat tissue physiologically predisposes to increased levels of OS [30]. Also 8OHdGuo values present a significative positive correlation with FM of subjects in agreement with the consideration that high fat percentage has been associated with high concentrations of OS by-products such as isoprostanes or the DNA oxidized and highly mutagenic base 8-oxo-7,8-dihydro-2′-deoxyguanosine [31].

Negative correlations were encountered analysing the relationship of pro-oxidant status with PA and TBW values. Probably the good state of health together with the fitness—assessed by the PA values in the subjects analysed—positively affects the metabolism, avoiding excessive radical production as hypothesized in other studies [18].

Inverse correlations resulted between DPPH with BMI and with TBW. The finding of an inverse correlation in this study of TBW with DPPH and d-ROM is supported—to our knowledge—by a single study in which the levels of glutathione peroxidase—the enzyme involved in the protection against ROS—show a direct association with the intracellular water (60% of TBW) [32].

### 3.2. Oxidative Status and Diet at Baseline (T_rc_)

A valuation of dietary habits of all participants was inferred by the questionnaire, and the percentages of self-reported frequencies of specific food consumption are reported in Table 4. A high intake of vegetables and fruits is appreciable for all subjects in line with Mediterranean diet indications and dietary habits resulted comparable between trained and untrained subjects.

In the literature observational evidence shows a negative association between levels of OS biomarkers and intake of vitamins and bioactive compounds contained in fruit and vegetables in healthy subjects [33,34]. Furthermore, as shown by several studies, vitamins can improve DNA repair enzymes, such as enzymes involved in DNA methylation or base excision repair. In this way, they contribute indirectly to decrease oxidative damage [35,36], as well as to the up-regulation of DNA repair mechanisms-related genes [37]. However, in this investigation no statistically significant relationships between biomarkers of OS and specific food intake frequencies were found.

### 3.3. Oxidative Status and Physical Activity in the Trained Group (at T_0_ and T_1_)

**Figure 2:**
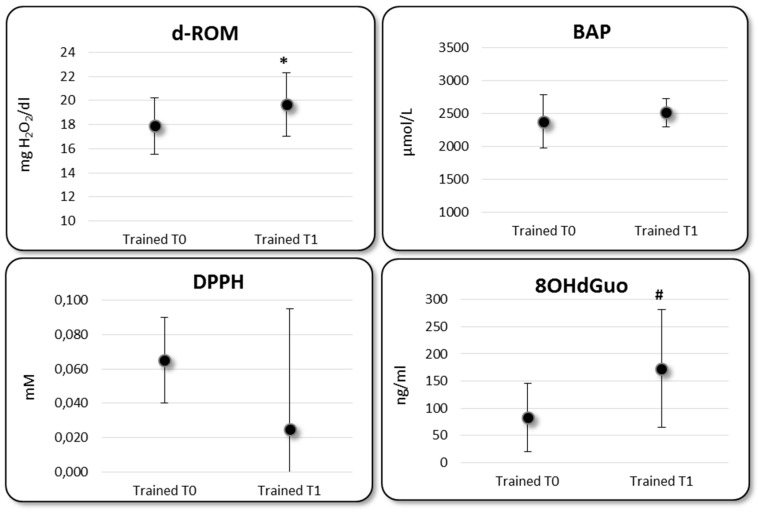
Biomarkers of OS status in trained subjects at T_0_ and T_1_ time (* *p* = 0.030 Trained T_0_ vs. T_1_; # *p* = 0.049 Trained T_0_ vs. T_1_).

The effect of physical exercise on the OS level was investigated comparing the OS bioindicators before (T_0_) and after (T_1_) a physical activity session in trained subjects (Figure 2): paired T test revealed statistically different means for d-ROM (*p* = 0.030) and 8OHdGuo (*p* = 0.049), with higher values at T_1_ time point. Referring to the two bio-indicators of FRAS, the mean value of BAP and DPPH resulted not statistically different between T_0_ and T_1_.

The literature affirms that acute aerobic and anaerobic exercises can elicit OS through excess exercise induced ROS production due to higher metabolic rate [38,39]. Studies observed that exercise-induced ROS are transient and this finding has been related to the induction of many redox-specific health adaptations [6]. For example the adaptation of the body’s antioxidant defence system is a result of exercise training [5,40,41]. Considering that the physical activity in the present study is intended as constant activity in accustomed trained subjects, the OS levels were found to increase, while remaining within the normal range, reinforcing the adaptation hypothesis. We detected a significant increase of OS measuring d-ROMs and 8OHdGuo after the training session. This may indicate that the fitness exercise performed by the trained group was enough to cause an increase of ROS production exceeding the antioxidant defences. Previous studies suggested that exercise must be exhaustive to overwhelm endogenous antioxidant defences and promote a state of OS [42]; however, this condition seems to be unnecessary, as confirmed in a review in which numerous studies showing significant increases of OS biomarkers with non-exhaustive exercise are reported [39].

The regular practice of physical activity associated with a balanced diet can be an important factor of health promotion, as also emerged in our study by diet investigation. A proper intake of vitamins and other antioxidant substances cooperates with the FRAS naturally present at systemic level. The trend of the two bioindicators of FRAS (BAP and DPPH) in this study suggests that the physical training-induced damage due to increased ROS was minimized by the increase of plasma antioxidant capacity favoured by other different endogenous or diet-introduced antioxidant substances.

## 4. Conclusions

The study revealed a redox balance ranging within the reference levels and optimal impedance values in all participants, highlighting a good health status of the study group, especially regarding the specific age range considered. This result may be attributable to the correct lifestyle followed by the subjects for all aspects considered as revealed also by the questionnaire analysis; in these subjects the physiological changes linked to modified or loss of age-related body functionalities seem to be well contrasted both by dietary habits and by regular physical exercise. In fact, the analysis of the frequency of consumption of the different food groups attests reasoned diet habits, rich of fruit and vegetables, and in line with the recommendations of public authorities to improve the physical and mental wellbeing. Within this group of healthy adults with homogenous lifestyles, differences of OS measured by d-ROMs, BAP, DPPH and 8OHdGuo appear to be modest and not statistically significant for all subjects. The quantified biomarkers are transient chemical species and they derives from a variety of metabolic pathways, making them unspecific markers of OS status. Evidence from studies exploring the effect of dietary patterns, suggests that beneficial effects of diet on OS may be related to the synergistic action of different dietary compounds rather than single food or bioactive substance effect.

Referring to physical activity no significant differences have been found between trained and untrained subjects, mainly due to the low number of participants in the untrained group. Another important consideration is that by the analysis of the questionnaire subjects classified as untrained it resulted they were not really sedentary: in fact some of them were walkers, others went to work by bike, etc., even if they didn’t practice regular and structured physical activity. Moreover they followed a healthy lifestyle (i.e., Mediterranean diet) like the trained subjects. Finally in the trained group physical exercise represents a significant contributor of OS, especially with reference to d-ROMs and 8OhdGuo values. Further research with more specific biomarkers of OS status should help clarify the role of different intensity exercise in the up-regulation of pro-oxidant/anti-oxidant balance and other redox-mediated health adaptations.

## Figures and Tables

**Table 1:** ANOVA analysis of the impedance measures stratified by selected individual characteristics of the study population (ns = not significant).

	*N*	Mean ± SD			
		BMI	PA (Degrees)	TBW (%)	FM (%)
**Gender**					
♂	32	24.2 ± 3.7	7.1 ± 0.7	26.1 ± 3.1	7.2 ± 3.9
♀	19	20.7 ± 2.8	6.2 ± 0.7	20.2 ± 1.7	6.5 ± 3.4
*p*		<0.001	≤0.0001	≤0.0001	ns
**Age**					
≤39	16	22.5 ± 4.7	7.0 ± 0.7	23.6 ± 4.0	6.4 ± 4.4
40–44	19	23.0 ± 3.7	6.6 ± 0.8	24.4 ± 4.0	7.5 ± 3.5
≥45	16	23.0 ± 3.0	6.8 ± 0.9	23.8 ± 3.8	6.9 ± 3.4
*p*		ns	ns	ns	ns
**Smoking habits**					
No	41	22.9 ± 3.9	6.7 ± 0.8	23.9 ± 4.03	7.1 ± 3.8
Yes	10	22.8 ± 3.4	6.9 ± 0.6	24.0 ± 3.5	6.3 ± 3.7
*p*		ns	ns	ns	ns
**Physical activity**					
Untrained	16	22.9 ± 3.7	6.8 ± 0.7	24.1 ± 3.6	6.7 ± 3.9
Trained	35	22.8 ± 3.9	6.7 ± 0.8	23.8 ± 4.1	7.0 ± 3.7
*p*		ns	ns	ns	ns

**Table 2:** Descriptive analysis of the OS parameters at rest condition of the whole group of participants (T_rc_ time point) stratified by gender, environmental contributions and life styles (ns = not significant).

		**d-ROMs** **(mg H_2_O_2_/dL)**		**BAP** **(µM)**		**DPPH** **(mM)**		**8OHdGuo** **(ng/mL)**
**Gender**	*N*		*N*		*N*		*N*	
♂	32	16.67 ± 3.99	32	2346.56 ± 631.05	31	0.07 ± 0.01	32	82.25 ± 139.08
♀	19	19.94 ± 3.88	19	2374.37 ± 167.53	19	0.07 ± 0.01	19	46.50 ± 54.93
*p*		<0.01		ns		ns		ns
**Physical activity level**	*N*		*N*		*N*		*N*	
Untrained	16	17.89 ± 4.11	16	2313.81 ± 163.65	15	0.07 ± 0.01	16	39.01 ± 55.31
Trained	35	17.89 ± 4.33	35	2376.63 ± 604.26	35	0.07 ± 0.01	35	82.61 ± 133.11
*p*		ns		ns		ns		ns
**Traffic level**	*N*		*N*		*N*		*N*	
Low	25	17.15 ± 3.43	25	2413.44 ± 671.79	24	0.07 ± 0.01	25	36.36 ± 40.55
Heavy	26	18.60 ± 4.83	26	2302.58 ± 273.62	26	0.07 ± 0.01	26	100.26 ± 152.01
*p*		ns		ns		<0.05		<0.05
**Smoking habits**	*N*		*N*		*N*		*N*	
No	41	17.59 ± 4.26	41	2390.49 ± 536.23	40	0.073 ± 0.01	41	76.18 ± 126.36
Yes	10	19.14 ± 4.04	10	2219.30 ± 353.11	10	0.07 ± 0.01	10	39.23 ± 46.68
*p*		ns		ns		ns		ns

**Table 3:** Bivariate correlations between body features and OS parameters. Spearman’s Rho coefficients and *p* values are reported only for significant correlations (ns = not significant).

	Spearman’s Coefficient; *p* Value			
	d-ROMs (mg H_2_O_2_/dL)	BAP (µM/L)	DPPH (mM)	8OhdGuo (ng/mL)
**Age**				
♂ (*N* = 32)	ns	ns	ns	ns
♀ (*N* = 19)	ns	ns	ns	ns
All (*N* = 51)	0.278; *p* < 0.05	ns	ns	ns
**BMI**				
♂ (*N* = 32)	ns	ns	ns	ns
♀ (*N* = 19)	ns	ns	−0.631; *p* < 0.005	ns
All (*N* = 51)	ns	ns	−0.509; *p* <0.0001	ns
**PA**				
♂ (*N* = 32)	−0.529; *p* < 0.001	ns	ns	ns
♀ (*N* = 19)	ns	ns	ns	ns
All (*N* = 51)	−0.557; *p* < 0.0001	ns	ns	ns
**TBW**				
♂ (*N* = 32)	ns	ns	ns	ns
♀ (*N* = 19)	ns	ns	ns	ns
All (*N* = 51)	−0.315; *p* < 0.05	ns	−0.323; *p* < 0.05	ns
**FM**				
♂ (*N* = 32)	Ns	ns	ns	ns
♀ (*N* = 19)	Ns	ns	ns	ns
All (*N* = 51)	0.306; *p* < 0.05	ns	ns	0.308; *p* < 0.05

**Table 4:** Frequency percentage of specific food intake of all 51 subjects.

	%		
	Never–1 time/week	2–4 times/week	5–7 times/week
**Alcoholic beverages**			
red wine	27.5	0.0	72.5
other alcoholic beverages	27.5	0.0	72.5
**Cooking methods**			
boiled	58.8	25.5	15.7
steam	41.2	35.3	23.5
fried	17.6	80.4	2.0
grilled	54.9	23.5	21.6
**Antioxidant foods**			
blueberries	54.9	45.1	0.0
cabbage	15.7	74.5	9.8
spinach	19.6	66.7	13.7
beet	62.7	33.3	3.9
blackberries	45.1	54.9	0.0
plum	33.3	62.7	3.9
cabbage	35.3	54.9	9.8
grapefruit	45.1	45.1	9.8
strawberries	5.9	76.5	17.6
orange	9.8	58.8	31.4
pepper	9.8	78.4	11.8
kiwi	25.5	52.9	21.6
beans	9.8	64.7	25.5
cauliflower	7.8	74.5	17.6
**Other foods**			
red meat	7.8	86.3	5.9
sausages	3.9	96.1	0.0
fish	5.9	94.1	0.0
eggs	3.9	96.1	0.0
fruits	0.0	17.6	82.4
vegetables	3.9	25.5	70.6
legumes	0.0	96.1	3.9
milk dairy	11.8	0.0	88.2
sugary beverages	33.3	64.7	2.0
dessert	5.9	66.7	27.5
junk food	47.1	52.9	0.0
coffee, tea	5.9	3.9	90.2
dark chocolate	25.5	66.7	7.8
